# Blood Glucose Level, Langerhans Pancreas and Lipid Profile of Diabetic Rats After Administration of Red Betel, Ginger and Cinnamon Combination Extract

**DOI:** 10.21315/tlsr2023.34.1.3

**Published:** 2023-03-31

**Authors:** Mega Safithri, Maria Bintang, Syaefudin

**Affiliations:** Department of Biochemistry, Faculty of Mathematics and Natural Science, IPB University, Dramaga Campus, Bogor 16680, Indonesia

**Keywords:** Red Betel, Rat Blood Glucose Level, β Cell, Langerhans Pancreas

## Abstract

Previous studies had reported antihyperglycemic activity *in vitro*, *in vivo* and *in silico* of red betel (*Piper crocatum*) extract, which was associated with its polyphenolic, tannins, alkaloids, and flavonoids compounds. This study aimed to determine blood glucose level, Langerhans pancreas, lipid profile and bodyweight of streptozotocin-induced diabetic rats after administrating red betel combination extract. Red betel combination extract is composed of red betel extract that was combined with ginger and cinnamon extracts. 16 male rats (*Sprague Dawley*) were divided randomly into two controls groups (Normal and diabetic groups; orally administered with 2 mL of aquadest for 14 days) and two extract groups (diabetic groups; orally administered with red betel combination extract 9 mL/kg BW and 13.5 mL/kg BW). Results showed that the administration of red betel combination extract for 14 days (9 mL/kg BW dosage) decreased the rat’s blood glucose level up to 55.42%, which was significantly different (*p* < 0.05) compared to the rat’s blood glucose levels on day 3. While the combination extract (dosages 9 mL/kg BW and 13.5 mL/kg BW) increased the numbers of rat Langerhans islets up to 10.9%–30.6%. The level of rat’s blood high-density lipoprotein (HDL) and triglyceride levels in the diabetic control group were significantly different (*p < 0.05*) compared to that of the diabetic with red betel combination extract and normal groups. The treatment orally with red betel combination extract (various dosages) for 14 days suppressed the weight loss of rats by 10%–11%.

**Highlights:**

- Red betel combination extract is composed of red betel extract that was combined with ginger and cinnamon extracts.
- The administration of red betel combination extract for 14 days (9 mL/kg BW dosage) decreased the rat’s blood glucose level up to 55.42% and also increased the number of rat Langerhans islets.
- The level of rat’s blood HDL and triglyceride levels in the diabetic control group were significantly different (*p* < 0.05) compared to that of the diabetic with red betel combination extract and normal groups.

## INTRODUCTION

Red betel *(Piper crocatum Ruiz & Pav)* is a bush plant originally from Peru and has spread to several regions of the world, including Indonesia. The stems of this plant are typically broad and segmented, 5 cm–10 cm apart with roots growing at each node. Moreover, a betel leaf has a stem with an elliptical, pointed, sub-acute at the base with a tapered tip, flat edge, shiny or non-administrative (Windono & Parfati 2016). Red betel plant is known to contain active compounds such as flavonoids, alkaloids, tannins, polyphenolic compounds and essential oils (Wulandari *et al*. 2018). Hence, it is often used for traditional alternative medicine (Lister *et al*. 2020). In addition, red betel been shown to have antihyperglycemic activity *in vivo* and could reduce blood glucose levels of diabetic rats up to 38.95% (Hasibuan *et al*. 2016). Antihyperglycemic activity *in vitro* showed that ethyl acetate fraction of the red betel has an IC_50_ value of 743.80 μg/mL with a competitive inhibitory mechanism to the alpha(α)-glucosidase (Weni 2018). In addition, red betel ethanol extract exhibited a competitive inhibition for α-glucosidase, similar to acarbose on α-glucosidase inhibition (Alfarabi *et al*. 2021). Further investigation using *in silico* method indicated that the active compound inhibiting α-glucosidase enzyme was columbine (Weni *et al*. 2020). Stilbene, phenol, linolenic acid, phytosteroid and α-tocopherol are bioactive compounds contained in the ethanol extract of red betel leaves. Stilbene and phenol have α-glucosidase inhibitory activity (Alfarabi *et al*. 2021). Antihyperglycemic activity of red betel leaves consumption may be due to the presence of its phytochemicals, including flavonoids, alkaloids and tannins (Safithri *et al*. 2016). Red betel extract has a bitter taste (Safithri *et al*. 2016). Therefore, by combining ginger and cinnamon extracts to red betel extract should be a better option in order to achieve a more suitable flavour and increase antihyperglycemic activity.

A supporting ingredient in Java tea functional drink is ginger extract *(Zingiber officinale)*, which has been investigated for its antihyperglycemic activity in diabetic rats (Naibaho *et al*. 2019). For instance, studies shows that ginger supplementation for three months was able to stabilize glucose levels in type 2 diabetes mellitus patients (Shidfar *et al*. 2015). While consuming cinnamon (*Cinnamomum burmannii*) extracts will inhibit glucose absorption in intestinal cells, that is by inhibiting α-glucosidase enzyme (75.9%) (Safithri 2012). *C. burmannii* extract has been reported to increase antihyperglycemic activity in streptozotocin-induced Sprague Dawley diabetic rats (Hasibuan *et al*. 2016) and has beneficial effects for type 2 diabetes mellitus patients, such as lowering blood glucose levels (Siswandi *et al*. 2020). This study was conducted to observe diabetic rats after administering red betel combination extract (*P. crocatum*, *Z. officinale*, and *C. burmannii* extracts) on blood glucose levels, Langerhans pancreas, blood lipid profile and rat body weight.

## MATERIALS AND METHODS

### Preparation of Red betel, Ginger, and Cinnamon Extracts

The leaves of red betel (*P.crocatum*) and barks of cinnamon (*C. burmannii*) were collected from Tropical Biopharmaca Research Center (Trop BRC) located in Dramaga, Bogor City, West Java, Indonesia. While ginger (*Z. Officinale*) was collected from the Indonesian Medicinal and Aromatic Crops Research Institute (IMACRI) located in Tajur, Bogor City, West Java, Indonesia. In order to obtain a sample with a size of 40 mesh, the red betel leaves were sorted, washed, drained, and then cut into small pieces. Then, the leaves are left to dry in an oven at 50°C for three days. Finally, the leaves were blended and filtered (Safithri *et al*. 2020a). The total of 5 g of powdered red betel was added to 100 mL of distilled water (1:20), then boiled for 15 min in a closed container. Filter paper was used to filter the sample. The filtrate was added with distilled water until a total volume of 100 mL was reached. The method for cinnamon bark powder was the same as that for red betel leaves powder.10 g of cinnamon bark powder was added to 100 mL of distilled water (1:10 w/v), then boiled for 15 min in a closed container. The sample was filtered with a filter paper, and the filtrate was added to distilled water until a total volume of 100 mL was reached. The method for ginger extraction was the same as that for cinnamon bark extractions. The obtained solutions are then stored at 8°C before being used. The red betel extract was combined with the ginger and cinnamon extract in a ratio of 42%: 25%: 33% (Safithri *et al*. 2020b).

### Animal Study

Sixteen (16) male rats (*Sprague Dawley*) were divided randomly into two controls groups and two extract groups. There were four rats in each group of treatment. First, the normal control group was intraperitoneally injected with NaCl 0.9% and orally administered with aquadest 2 mL for 14 days. Second, the diabetic control group was intraperitoneally injected with streptozotocin (STZ) 50 mg/kg BW and orally administered with aquadest 2 mL for 14 days. Thirdly, the red betel group was intraperitoneally injected with STZ 50 mg/kg BW and orally administered with red betel combination extract 9 mL/kg BW. Fourth, similar to that of the third group but with the administration of 13.5 mL/kg BW red betel combination extract. Treatment with extract was started 48 h after streptozotocin injection. Animal use for this research has been approved by The Institutional Animal Ethics Committee (PT. Bimana Indomedical) with a code of R.02-11-IR (Windari *et al*. 2019).

### Fasting Blood Glucose Level

Blood samples were collected from the tail veins of rats that had been fasting for 18 h. An electronic glucometer kit was used to monitor glucose levels before the STZ injection (on day 0) and after the STZ injection on days 2, 9 and 16. The kits used to measure blood insulin levels are a single touch GlucoDr kit (Mercodia, Sylveniusgatan, Uppsala) and a microplate reader (BioRad 3550) with the enzyme linked immunosorbent assay (ELISA) (Hasibuan *et al*. 2016). Blood glucose levels were measured in mg/dL and recorded in percentage changes.

### Changes of Rat Body Weight

The body weight of rats was observed weekly during the experiment. Data were measured in percentage changes.

### Measurement of Blood Lipid Level

The Photometer 5010 (Robert Riele GmbH & Co KG, Berlin, Germany) was used to measure blood lipid levels. Meanwhile, a commercial kit (Roche Diagnostics GmbH, Mannheim, Germany) was used to analyse triglycerides, total blood cholesterol and HDL level (Hasibuan *et al*. 2016).

### Pancreas Immunohistopathological Examinations

Two rats from each group were injected with xylazine 10 mg/kg BW, euthal 200 mg/kg BW, and ketamine 80 mg/kg BW before necropsy on day 16. Immuno-histopathological examination was performed on the pancreas organ (Hasibuan *et al*. 2016).

### Eosin Hematoxylin Staining

Rat’s pancreatic tissue was soaked separately in xylol, alcohol 95% and alcohol 70%. After that, the tissue was washed with running water and then soaked in Mayer’s hematoxylin dye reagent for 5 mins before being rewashed. Next, the acquired product was alternately soaked in an acid alcohol solvent, ammonia solution, and eosin dye. Later on, alcohol 95%, alcohol 99.8% and xylol, were subsequently used to wash the rat’s pancreatic tissue. Finally, the number of Langerhans islets in rats’ pancreatic tissue was counted under a light microscope. A Nikon Eclipse 80i DS Fi1 was used to take photomicrographs of the rat’s pancreatic tissue (Hasibuan *et al*. 2016).

### Immunohistochemical Staining

The immunohistochemical staining stage started from the histopathological preparations. After deparaffination and rehydration, the preparation was soaked in running water for 5 min, then soaked in distilled water for 5 min and in 1 mL of 30% H_2_O_2_ for another 5 min, and then soaked again in distilled water for 5 min. Afterwards, the preparation was soaked in citrate buffer at temperature 100°C for 20 min, then soaked in running water for 5 min and soaked in distilled water for another 5 min, and then soaked in phosphate-buffered saline (PBS) two times for 2 min each. After that, the preparation was dripped with 20 mL–30 mL of blocking protein and left at room temperature for 15 min. And then, the preparation was dripped with 20 mL–30 mL of primary antibody and incubated for 60 min, then the preparation was soaked in PBS 3 times for 2 min each, then given 20 mL–30 mL trakkie universal link solution and incubated for 20 min. After that, the preparation was soaked in PBS three times for 2 min each, then Trek-avidin HRP was added to the preparation and incubated for 10 min, afterwards the preparation was soaked in PBS three times for 2 min each. After that, the preparations were given 20mL–30 mL of DAB dye and incubated for 2 min–3 min, then soaked in distilled water for 5 min, and stained with Hematoxylin for 15 sec, then sequentially soaked in distilled water for another 5 min, in 95% alcohol two times for 30 sec each and in 100% alcohol two times with 10 dips each, then soaked in xylol three times for 15 min each (Biocare Medical 2011). After the staining process was completed, the microscope slide was dried and dripped with 3-aminopropyltriethoxycilen adhesive and then covered with an object glass, then the preparation was labelled and ready to be observed under the light microscope. Observation of immunohistochemical staining preparations was to count the number of pancreatic β-cells that counted from 4 islets of Langerhans per preparation.

The number of pancreatic β-cells was counted using immunohistochemical staining method. In the first stage, the histopathology preparation was done before staining. In the second stage, the rat pancreatic tissue was deparaffinised and rehydrated. In the third stage, the rat pancreatic tissue was soaked in a phosphate buffer solution (PBS) for 2 min. In the fourth stage, the tissue was incubated for 60 min after it was dropped with inhibitory protein, and it was added with rat monoclonal anti-insulin antibody. Finally, the acquired product was soaked in PBS and added with a secondary antibody, i.e., Trek Universal Link solution then resoaked in PBS and added with Trek-Avidin-HR. Furthermore, the preparation was visualised using 1.3-diaminobenzidine (DAB), soaked in distilled water for 5 min, and then stained with hematoxylin dye. The last stages were dehydration, clearing, mounting, and observation under the light microscope (Hasibuan *et al*. 2016). The one-way analysis of variance (ANOVA) was used for statistical analysis in this research with significant differences between means were compared by The Tukey T Test at *p* < 0.05. All data were expressed as mean ± SD (Windari *et al*. 2019).

## RESULTS

### Rat Blood Glucose Level

The effect of feeding red betel combination extract in diabetic rats showed that blood glucose levels in all groups on day 0 (before STZ induction) were in a normal range (90 mg/dl–100 mg/dl) (Table 1). All of the groups were not significantly different (*p* > 0.05). On day 2, the rat’s blood glucose increased up to 2 folds–3 folds after STZ induction (50 mg/kg BW) (Table 1), significantly different (*p* < 0.05) compared to those for NaCl 0.9% induction group.

The blood glucose levels of rats given various dosages of red betel combination extract were decreased by 18 to 55% after 14 days of treatment compared to the diabetic control group given aquadest. Blood glucose levels of rats decreased up to 55.42% after administration of red betel combination extract for 14 days (9 mL/kg BW dosage). It was significantly different (*p* < 0.05) compared to the rat’s blood glucose levels on day 3 (Table 1). However, administering red betel combination extract for 14 days (13.5 mL/kg BW dosage) decreased the rat’s blood glucose level up to 18.62%. It was not significantly different (*p* > 0.05) compared to the rat’s blood glucose levels on day 3 (Table 1). It indicates that red betel combination extract had antihyperglycemic activity.

### Rat Langerhans Islet and **β**-cells

The administration of red betel combination extract for 14 days with various dosages (9 mL/kg BW and 13.5 mL/kg BW) increased the numbers of rat Langerhans islets up to 10.9%–30.6% (Table 2, Fig. 1). Meanwhile, the administration of orally aquadest 2 mL for 14 days in the diabetic control group (STZ induction) decreased the numbers of rat Langerhans islets up to 67.4%. The results indicate that red betel, ginger, and cinnamon’s bioactive compounds could repair rat Langerhans islets, releasing the insulin hormone from pancreatic β-cells. Therefore, blood glucose was absorbed into the cells, and blood glucose level was reduced.

### Rat Blood Lipid Profile

The blood lipid profile of the diabetic rats was measured after 14 days of oral treatment of a combination red betel extract or aquadest. The rats’ total blood cholesterol levels in all groups were not significantly different (*p* > 0.05) (Table 3). However, the level of rat’s blood HDL in the diabetic control group (orally administered with aquadest) was significantly different (*p* < 0.05) compared to that of the diabetic + red betel combination extract and normal groups (Table 3). Thus, the rats’ blood triglyceride levels in the diabetic control group (orally administered with aquadest) were significantly different (*p* < 0.05) compared to that of the diabetic + red betel combination extract and normal groups (Table 3). The results showed that the red betel combination extracts could increase the blood HDL and decrease blood triglyceride levels in rats to a normal level.

### Rat Body Weight

On day 0, the rats’ body weight in all groups was not significantly different (*p* > 0.05) (Table 4). However, on days 9 and 16, after streptozotocin induction, the rats’ body weight in diabetic + red betel combination extract groups decreased. Except for the normal group, the rat’s body weight increased. The highest rat bodyweight reduction (17.49%) was shown in the diabetic + aquadest group. The treatment orally of red betel combination extract (various dosages) for 14 days suppressed the weight loss of rats by 10%–11%. It indicated that the treatment of red betel combination extract was able to maintain the diabetic rat’s body weight.

## DISCUSSION

The antihyperglycemic activity of red betel combination extract was higher than the red betel extract only or combination extract of red betel and cinnamon (Hasibuan *et al*. 2016). These results showed that the extracts of *Z. officinale* increased antihyperglycemic activity. The red betel bioactive compounds that play antidiabetic agents are flavonoids, alkaloids and tannins (Safithri *et al*. 2016). Columbin is one of the bioactive compounds in red betel that has stability and could inhibit α-glucosidase in silico (Weni *et al*. 2020). Bioactive compounds of *C. burmannii* consist of eugenol, coumarin and cinnamaldehyde (Budiastuti *et al*. 2020). *C. burmannii* extract could improve fasting blood glucose levels in diet-induced obese hyperglycemic mice by suppressing major regulatory genes in hepatic gluconeogenesis, phosphoenolpyruvate carboxykinase and glucose-6-phosphatase (Cheng *et al*. 2012). The main bioactive compounds of ginger include zingerone, gingerol, shogaol and paradol (Shidfar *et al*. 2015). Zingerone has antidiabetic activity (Ahmad *et al*. 2015). The glycemic indices in type 2 diabetes patients could be improved by supplementing ginger for 3 (three) months (Shidfar *et al*. 2015). Bioactive compounds contained in *Z. officinale* could increase the antihyperglycemic activity of Java tea beverage (Indariani *et al*. 2014).

Intraperitoneally streptozotocin induction can damage β-cells of pancreatic, and reduces insulin secretion and biosynthesis, resulting diabetes in rats (Szkudelski 2001). This study showed that the combination of red betel extract (9 mL/kg BW) for 14 days increased the number of β-cells and Langerhans islets of pancreatic to a normal level. This result is in line with Rekasih *et al*. (2021) research results, where diabetic rats were given an aqueous extract of java tea mixture (18.2 mL/kg BW) for 14 days, which showed enhancement of the number of pancreatic β-cells in the pancreatic Langerhans islets. In addition, this study showed that the red betel combination extract could increase the number of pancreatic β-cells to increase the rats blood insulin level, reducing blood glucose levels in diabetic rats.

In this study, the total cholesterol level in the blood serum of diabetic rats for 14 days did not significantly increase after STZ induction. This finding is similar to that reported by Hasibuan *et al*. (2016), indicating total cholesterol levels did not significantly increase on day 14 in diabetic rats after STZ at the dosage of 50 mg/kg BW induction. Treatment of diabetic rats with a combination of red betel extract for 14 days led to a significant reduction in triglyceride levels and a significant enhancement in HDL-cholesterol levels. The possible mechanism is enhanced insulin secretion from pancreatic β-cells, which further triggers the synthesis of fatty acid and triglycerides in adipose tissue and liver (Choudhari *et al*. 2017).

Bodyweight loss is one of the symptoms in diabetic rats, and several factors can cause it. It can be caused by the feed intake given being imbalanced with extreme hunger conditions (Windari *et al*. 2019). Administration of *P. crocatum* water extract (1350 mg/kg BW) for 14 days could repress weight loss in diabetic rats up to 4.90 % (Hasibuan *et al*. 2016). Inline, administration of the ethanol extract of *Toona sinensis* leaves (300 mg/kg BW) for 14 days showed repression of weight loss up to 0.37% in diabetic rats (Theresia *et al*. 2017). The administration of red betel combination extract at a dose of 9 mL showed better results than a higher dose of 13.5 mL in reduce blood glucose levels, increase the number of β-cells, Langerhans islets and the blood HDL, decrease blood triglyceride levels, and maintain the diabetic rat’s body weight. This result is in accordance with Hasibuan *et al*. (2016), that the treatment with small doses has a better effect than higher doses, in thus study the KDS 2 group was able to reduce their blood glucose levels up to 50.98% and increase their numbers of Langerhans islets up to 3.3-fold, which was much better than group KDS 3.

## CONCLUSION

Daily treatment with the various dosage of the red betel combination extract for 14 days indicated decreased blood glucose and triglyceride level, increased amount of rat Langerhans pancreas β-cell islets number and HDL level, and suppressed body weight loss in diabetic rats.
